# Aerobic Exercise Preserves Olfaction Function in Individuals with Parkinson's Disease

**DOI:** 10.1155/2016/9725089

**Published:** 2016-11-23

**Authors:** Anson B. Rosenfeldt, Tanujit Dey, Jay L. Alberts

**Affiliations:** ^1^Department of Biomedical Engineering, Cleveland Clinic, 9500 Euclid Ave., Cleveland, OH 44119, USA; ^2^Quantitative Health Sciences, Cleveland Clinic, 9500 Euclid Ave., Cleveland, OH 44119, USA; ^3^Center for Neurological Restoration, Cleveland Clinic, 9500 Euclid Ave., Cleveland, OH 44119, USA; ^4^Cleveland FES Center, L. Stokes Cleveland VA Medical Center, 10701 East Blvd, Cleveland, OH 44106, USA

## Abstract

*Introduction*. Based on anecdotal reports of improved olfaction following aerobic exercise, the aim of this study was to evaluate the effects of an 8-week aerobic exercise program on olfaction function in individuals with Parkinson's disease (PD).* Methods*. Thirty-eight participants with idiopathic PD were randomized to either an aerobic exercise group (*n* = 23) or a nonexercise control group (*n* = 15). The aerobic exercise group completed a 60-minute cycling session three times per week for eight weeks while the nonexercise control group received no intervention. All participants completed the University of Pennsylvania Smell Identification Test (UPSIT) at baseline, end of treatment, and a four-week follow up.* Results*. Change in UPSIT scores between the exercise and nonexercise groups from baseline to EOT (*p* = 0.01) and from baseline to EOT+4 (*p* = 0.02) favored the aerobic exercise group. Individuals in the nonexercise group had worsening olfaction function over time, while the exercise group was spared from decline.* Discussion*. The difference in UPSIT scores suggested that aerobic exercise may be altering central nervous system pathways that regulate the physiologic or cognitive processes controlling olfaction in individuals with PD. While these results provide promising preliminary evidence that exercise may modify the disease process, further systematic evaluation is necessary.

## 1. Introduction

The majority of individuals with Parkinson's disease (PD) experience olfaction dysfunction [1, 2]. Hyposmia and anosmia are associated with loss of enjoyment of food, difficulty managing body weight, safety concerns (i.e., detecting gas and smoke), insecurities with body odor, and social isolation [3]. These factors lead to a decreased quality of life and increased rates of depression when compared to individuals with normal sense of smell [4].

Although the exact mechanism for olfaction loss in Parkinson's disease is unknown, it is likely that olfaction dysfunction is due to changes in the central nervous system (CNS) and is not a result of damage to the peripheral olfactory system [5]. It has been proposed that olfaction dysfunction in Parkinson's disease evolves from Lewy bodies formed in the olfactory bulb and progresses to brain stem nuclei such as the locus coeruleus and substantia nigra and eventually to the cerebral cortex [6]. In addition, neurotransmitters such as acetylcholine, norepinephrine, serotonin, and, to a lesser extent, dopamine, all of which are typically altered in Parkinson's disease, impact olfaction through various direct and indirect pathways [7].

The clinical importance of hyposmia continues to evolve, and in individuals with PD olfaction testing can be used at a diagnostic tool [8] and is predictive of long-term cognitive decline and postoperative delirium [9–11]. In a large cohort of de novo patients, it was reported that PD patients with hyposmia exhibit more severe motor symptoms and required greater levodopa-equivalent at a 2.5-year followup compared to those patients with normal olfactory function [12]. The growing importance of olfaction as a diagnostic and predictive tool in individual with PD highlights the need for further examination.

While hyposmia is not a target of PD treatment per se, antiparkinsonian medications have no effect on olfaction dysfunction [13, 14]. It has been proposed that deep brain stimulation (DBS) may indirectly affect olfaction; however, most large randomized DBS studies have not utilized an olfaction outcome measure, and the few DBS studies that have reported an olfaction outcome have been conducted on relatively small sample sizes and have yielded conflicting results [15–18]. There is preliminary evidence that exercise may have a positive impact on olfaction. In an 8-week swimming intervention in adult rats, synapsin and neurotrophic factors in the olfactory bulb were greater in the exercise group than the nonexercise control group [19]. In a longitudinal study of over 1800 older adults, those who exercised three times per week were at a lower risk of developing olfaction dysfunction over a 10-year follow-up period [20]. These studies provide rationale to investigate the idea that exercise may facilitate neuroplasticity of the olfaction system.

Aerobic exercise, in animal models of Parkinsonism, has been shown to have neuroprotective and neurorestorative effects, likely through modulation of neurotrophic factors that support angiogenesis and synaptogenesis, suppress oxidative stress, and enhance mitochondrial function [21]. Recently, we have demonstrated that a specific mode of aerobic exercise, forced exercise (FE), reduces motor symptoms as measured by blinded clinical assessments, improves upper extremity motor functioning and control, and produces changes in cortical and subcortical functional connectivity [22–25]. In our preliminary study examining forced and voluntary rate cycling, some participants with PD self-reported improvements in olfaction following aerobic exercise, thus leading to the hypothesis that aerobic exercise may be facilitating neuroplasticity within the olfactory system. The aim of this project was to formally evaluate the effects of an 8-week forced and voluntary aerobic exercise program on olfaction function in individuals with Parkinson's disease.

## 2. Methods

### 2.1. Participants

Thirty-eight individuals with a diagnosis of idiopathic PD completed the informed consent process approved by the Cleveland Clinic Institutional Review Board. Primary inclusion criteria were clinical diagnosis of idiopathic PD by a neurologist, age between 30–75 years, and Hoehn and Yahr stages II-III when off antiparkinsonian medication. Primary exclusion criteria were existing cardiopulmonary disease or stroke, presence of dementia, and any medical or musculoskeletal contraindications to exercise. Participants completed a cardiopulmonary exercise (CPX) test on a stationary bicycle equipped with MedGraphics CardiO2/CP system with Breeze software and a twelve-lead electrocardiograph to screen for cardiac abnormalities that may warrant exclusion from the study.

### 2.2. Outcome Measure

The University of Pennsylvania Smell Identification Test (UPSIT), a 40-item “scratch and sniff” test, has been established as a valid and reliable tool for individuals with olfaction dysfunction and healthy controls [26]. After scratching the scent area, the participant selects a smell from 4 options in a forced-choice paradigm. A higher score (out of 40 points) indicates better odor identification. The UPSIT is a self-administered test that is objectively scored with an answer key. Testing was completed at baseline, end of treatment (EOT), and 4-week followup after end of treatment (EOT+4). In order to test participants in the off-medication state, subjects were asked to refrain from taking their PD medications after 8 pm the night before.

A blinded rater completed the Unified Parkinson's disease Rating Scale (UPDRS) motor examination, a standardized test that assesses motor function in individuals with PD.

### 2.3. Experimental Design

Following baseline testing, individuals were randomized into one of the following groups: (1) FE Cycling (FE), (2) Voluntary Exercise Cycling (VE), and (3) Nonexercise control group. Randomization was performed by having participants draw an envelope from a nonreplenished box. Of note, olfaction testing was added to the study testing protocol of an ongoing aerobic exercise study due to subjects' self-report of improved smell; thus the sample sizes were not evenly distributed.

### 2.4. Exercise Intervention

Participants in the VE and FE groups attended exercise sessions in the Neural Control Laboratory of the Cleveland Clinic, three times per week for a total of eight weeks. Participants were asked to take their PD medication as prescribed by their neurologist on the day of each exercise session. During exercise session, participants in the VE groups performed a 10-minute warm-up, 40- minute exercise set, and a 10-minute cool-down on a semirecumbent bike at a self-selected pace. Participants were encouraged to maintain a target heart rate zone of 60–80% based on heart rate reserve (HRR) method using results from individual maximal CPX test.

The FE group exercised for an identical period of time and target heart rate zone on a semirecumbent stationary exercise cycle custom engineered with a motor and accompanying control algorithm designed to augment the individual's torque production during pedaling, thus resulting in a steady, high-rate cadence. It is important to note that the FE approach required active participation from the participant and that cycling was not passive. The motor assisted the individual in achieving a pedaling rate 30% greater than their preferred voluntary rate as determined during CPX testing, a percentage increase that resulted in global motor improvements in our previous work with PD [22, 27]. For both exercise groups, cadence in revolutions per minute (rpm) and heart rate were recorded for each session.

The control group received no exercise intervention and was asked to continue their current level of physical activity.

### 2.5. Statistical Analysis

The primary outcome was the change in UPSIT scores from (1) baseline to EOT and (2) baseline to EOT+4. Shapiro-Wilk test was used to determine normality of the variables considered in the study. A 3 × 3 analysis of variance (ANOVA) model was used to examine UPSIT score changes at three time points (baseline, EOT, and EOT+4) for three groups (FE, VE, and nonexercise) and the interaction between the time and group variables. A two-sample *t*-test was performed to determine the influence of exercise performance variables, cadence and HRR, between the two exercise groups. An analysis of covariance (ANCOVA) model was used to determine the association between the UPSIT and the exercise performance variables, HRR and cadence. All hypothesis testing was completed at 5% level of significance.

## 3. Results

Using UPSIT score as the dependent variable in the ANOVA model, neither group (*F*_2,105_ = 0.09, *p* = 0.91), time (*F*_2,105_ = 0.30, *p* = 0.74), nor the interaction between group and time (*F*_4,105_ = 0.24, *p* = 0.92) was significant. While a trend was present for the VE group to be exercising at a greater intensity as measured by HRR, there was no significant difference between exercise intensity for the VE and FE groups with means of 57.9 and 48.9 percent of HRR, respectively (*p* = 0.06). Results from the ANCOVA, using HRR as the dependent variable, revealed a nonsignificant interaction effect between HRR and changes in UPSIT scores between VE and FE (*p* = 0.48 at EOT; *p* = 0.51 at EOT+4). There was a significant difference in cadence between the VE and FE groups, with means of 69.7 and 82.9 rpms, respectively (*p* < 0.01); however, an ANCOVA model, using cadence as the dependent variable, revealed a nonsignificant interaction between cadence and change in UPSIT score between groups (*p* = 0.13 at EOT; *p* = 0.59 at EOT+4). Due to the similarities in exercise performance variables, data were collapsed across exercise groups for comparison to the nonexercise control group. Baseline demographics, provided in Table 1, were similar between the exercise and the nonexercise groups.

**Figure 1:**
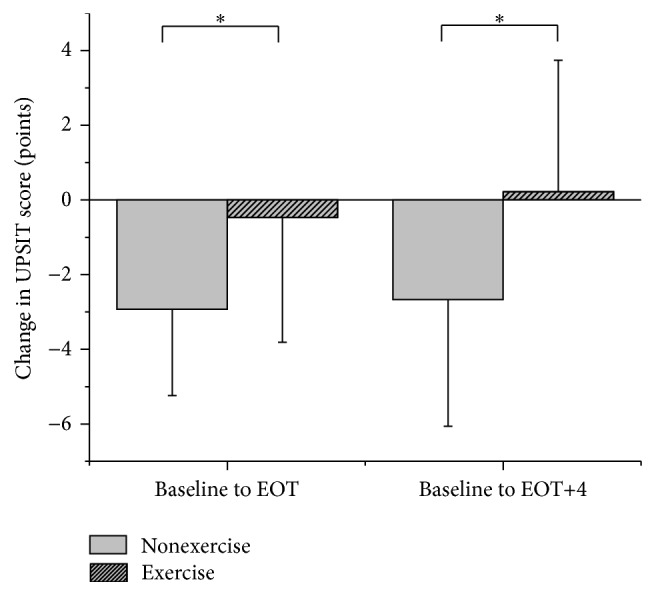
Mean change in UPSIT scores from baseline to EOT. There was a significant difference (indicated with *∗*) between the exercise and nonexercise groups in change in UPSIT scores from baseline to EOT and EOT+4, respectively. A positive change in UPSIT score indicates improved odor identification.

**Figure 2:**
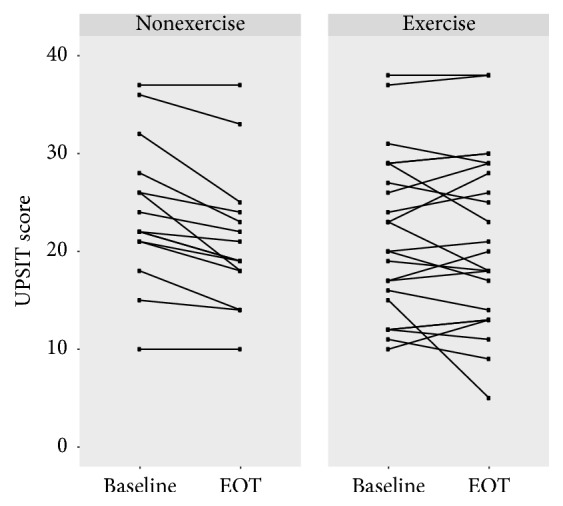
UPSIT scores at baseline and EOT for individuals in the exercise and nonexercise groups. At EOT, no participants in the nonexercise group demonstrated an improvement in UPSIT score; in contrast, 12 out of 23 individuals in the exercise group displayed an improvement in UPSIT score. Of note, 2 sets of individuals in the exercise group and 1 set of individuals in the nonexercise group scored identically from baseline to EOT; thus the lines are overlapping.

Table 2 and Figure 1 provide summary statistics for the exercise and control groups. A *t*-test indicated a significant difference in UPSIT scores between exercise and nonexercise groups from baseline to EOT (*p* = 0.01) and from baseline to EOT+4 (*p* = 0.02).  Figure 2 is a graphical depiction of the individual responses from each the groups from baseline to EOT. At EOT, no participants in the nonexercise group demonstrated an improvement on the UPSIT with a mean decrease of 2.9 (2.3) points. In contrast, 12 out of 23 individuals in the exercise group demonstrated an improvement in UPSIT scores; overall there was a mean decrease of 0.5 (3.3) points. From baseline to EOT+4, the nonexercise group had a decrease of 2.7 (3.4) points in UPSIT score while the exercise group exhibited a slight improvement of 0.2 (3.5) points.

There was no relationship between responders (those who improved their UPSIT score) and nonresponders (those who stayed the same or got worse in their UPSIT score) and the demographic variables listed in Table 1.

## 4. Discussion

Based on the UPSIT data, PD patients who did not exercise demonstrated a worsening of olfaction throughout the 8-week study and 4-week follow-up period, while those participating in aerobic exercise were spared from further worsening of olfaction function. The significant difference in UPSIT scores between the exercise and nonexercise groups suggests that aerobic exercise may be altering neurophysiological pathways or neurotransmitter function that regulate the physiologic or cognitive processes controlling olfaction [19, 20]. While we are not able to determine the exact mechanism underlying a sparring of olfaction, it is plausible, based on results from animal exercise studies, that the physiological changes (i.e., increased neurotrophins, neurotransmitters, and improved functional connectivity) and increases in cerebral blood flow associated with intensive aerobic exercise may have facilitated function of the olfaction system centrally or improved the higher level cognitive processes associated with odor detection [21, 28, 29]. While our previous imaging data supports altered CNS patterns of activation in the primary motor cortex, supplementary motor area, thalamus, globus pallidus, and putamen [24, 25], there is still much unknown about the role that aerobic exercise plays in modifying the structural and functional role of the CNS.

Although debated, olfaction function may worsen with disease duration [30], which is consistent with the nonexercise group demonstrating a decline in UPSIT scores over time. The wide range of change in UPSIT scores from the exercise group gives rise to the possibility that there is an individualized neurophysiological response to exercise ([28, 31, 32]). Individualized responses are reported with pharmacological interventions to PD, where some individuals exhibit a strong favorable response to levodopa therapy and others experience modest benefits [33]. Since our previous research revealed acute bouts of FE that resulted in CNS changes similar to those seen with Parkinson's disease medications [24, 25], it is possible that, similar to medication, individuals experience varying responses to aerobic exercise. The genetic response to exercise continues to be evaluated; Bath and colleagues reported impaired odor discrimination associated with brain derived neurotrophic factor (BDNF) val66met polymorphism in mice and propose a mechanism of decreased neurogenesis in the olfactory bulb as a result of the polymorphism [34]. While we are unable to speculate if genetics played a role in our results, the role of genetics in response to exercise in individuals with PD is an area for future study.

There are limitations to the current study. First, the sample was a relatively small group of individuals with mild to moderate Parkinson's disease; thus the data should be interpreted within this context. A larger scale (*N* = 100) clinical trial is currently testing a similar cycling protocol that includes a variety of motor and nonmotor outcomes, including the UPSIT. Second, we did not screen for individuals who may have had preexisting nasal disease or olfactory dysfunction. Third, although the UPSIT is a well-studied test, the minimal clinical important difference is unknown; thus we are not able to determine if a change in the score is meaningful to the participant. Additionally, although the UPSIT is an odor identification test that is easily administered in a clinical setting, odor detection and threshold are not measured by this test. Notably, there was no difference in UPSIT scores between the FE and VE groups; thus it appears that the mode of cycling was less important than the aerobic nature of the exercise. In the future it will be important to determine the relationship between mode, frequency, duration, and intensity of aerobic exercise and olfaction dysfunction in PD.

These findings, although preliminary, have potential to impact quality of life in individuals with PD. Hyposmia is one of the top five symptoms in individuals diagnosed with PD ≤6 years in duration [35], and individuals with olfactory dysfunction are more likely to report difficulties with activities of daily living and to rely on community resources [36]. A meaningful implication of halting the progression of anosmia with aerobic exercise is the potential that exercise may modify the disease progression. The difference in UPSIT scores exhibited by the exercise group supports previous findings that intensive aerobic exercise is linked to global changes in PD function [22, 23]. This work may have significant implications regarding the relationship between exercise and brain function and the potential to modify the course of this progressive neurological disorder through exercise.

## 5. Conclusion

In this study, individuals with PD who participated in 24 sessions of aerobic exercise maintained their olfaction function as measured by the UPSIT, while individuals who did not exercise demonstrated a worsening in UPSIT scores. While these results provide promising preliminary evidence that exercise may modify the disease process, further systematic testing is needed.

## Figures and Tables

**Table 1:** Participant baseline demographics.

	Nonexercise	Exercise (VE + FE)	*p* value
Sample size	15	FE = 9 VE = 14	
Age, years (SD)	60.9 (7.2)	60.5 (7.4)	0.85
Gender, male	8	16	0.33
Disease duration, years (SD)	3.3 (3.1)	3.3 (2.1)	0.93
Baseline UPSIT, points (SD)	24.0 (7.3)	21.6 (8.2)	0.35
Baseline UPDRS motor examination, points (SD)	21.9 (5.5)	23.5 (9.9)	0.52

FE: forced exercise; SD: standard deviation; UPDRS: Unified Parkinson's Disease Rating Scale; UPSIT: University of Pennsylvania Smell Identification Test; VE: voluntary exercise.

**Table 2:** Summary statistics for change in UPSIT scores from baseline to EOT and EOT+4.

	Mean of change in UPSIT (points)	Standard deviation (points)	Range (points)	*p* value
Baseline to EOT				
Nonexercise	(2.9)	2.3	(8.0)–0.0	
Exercise	(0.5)	3.3	(10.0)–5.0	0.01
Baseline to EOT+4				
Nonexercise	(2.7)	3.4	(10.0)–4.0	
Exercise	0.2	3.5	(7.0)–8.0	0.02

( ) indicates a score indicating a worsening in UPSIT score compared to baseline. A positive number indicates an improvement in UPSIT score.

EOT: end of treatment; EOT+4: end of treatment + 4 weeks; UPSIT: University of Pennsylvania Smell Identification Test.
